# Experiences of mental health nurses who give nursing intervention among child and adolescent with cyberbullying: a qualitative study

**DOI:** 10.1186/s12912-025-03182-x

**Published:** 2025-05-14

**Authors:** Iyus Yosep, Ai Mardhiyah, Suryani Suryani, Rohman Hikmat, Kurniawan Kurniawan, Heni Purnama

**Affiliations:** 1 Department of Mental Health, Faculty of Nursing, Universitas Padjadjaran, Jl. Raya Ir. Soekarno KM. 21 Hegarmanah, Jatinangor, Sumedang, West Java 45363 Indonesia; 2 Department of Pediatric Nursing, Faculty of Nursing, Universitas Padjadjaran, Jawa Barat, Sumedang, Indonesia; 3 Nursing Department, Faculty of Health Science, Universitas ‘Aisyiyah Bandung, Bandung, Indonesia; 4 School of Nursing, STIKEP PPNI Jawa Barat, Jawa Barat, West Java Indonesia

**Keywords:** Adolescents, Cyberbullying, Mental health nurses

## Abstract

**Background:**

The incidence of cyberbullying among adolescents has been increasing significantly each year, causing negative psychological impacts such as anxiety, low self-esteem, and depression. Mental health nurses play a pivotal role in mitigating these effects and supporting adolescent victims of cyberbullying through comprehensive nursing care.

**Objective:**

This study aimed to investigate the experiences of Mental health nurses in providing nursing interventions to adolescent victims of cyberbullying, focusing on challenges, strategies, and specific care practices.

**Methods:**

A qualitative descriptive approach was employed, utilizing semi-structured interviews to gather data from 12 Mental health nurses working in child and adolescent rehabilitation units at a psychiatric hospital in West Java Province, Indonesia. Participants were selected using purposive sampling to ensure a diverse representation of nurses with experience in handling cases of cyberbullying. Data were analyzed thematically, with specific attention to the identification of recurring patterns and themes related to nurses’ interventions and experiences.

**Results:**

The analysis identified five main themes: (1) types of bullying experienced by adolescents, (2) factors influencing the impact of cyberbullying, (3) obstacles and difficulties faced by nurses, (4) specific nursing interventions employed, and (5) unique experiences and coping strategies of nurses. Nurses underscored the challenges of addressing the multifaceted effects of cyberbullying and the importance of individualized interventions to support victims effectively.

**Conclusion:**

The study highlights the critical role of mental health nurses in providing targeted, holistic care for adolescent victims of cyberbullying. Nurses emphasize the need for comprehensive care strategies, the involvement of parents, and collaboration with the adolescent’s social environment to foster positive coping mechanisms and resilience. These findings advocate for healthcare policies and training programs that empower nurses to effectively address cyberbullying and provide robust support to affected adolescents.

**Supplementary Information:**

The online version contains supplementary material available at 10.1186/s12912-025-03182-x.

## Introduction

Increased use of social media causes teenagers to engage in cyberbullying behavior. The greatest risk of internet abuse in adolescents is cyberbullying at 30.27%. In this study, cyberbullying receives the greatest percentage compared to other negative impacts [1]. Cyberbullying is an important problem because perpetrators use communication technology to threaten or harm other people, including sending messages in the form of threats of physical or psychological violence, spreading rumors, or statements that trigger problems in other people’s relationships via email, text messages, or social media [2, 3]. Cyberbullying involves actions intended to harm, embarrass, or distress others [4]. This behavior is typically repeated against victims who are unable to defend themselves, carried out by individuals or groups using electronic media [5].

Acts of cyberbullying can be influenced by personal factors and situational factors. Personal factors include internet addiction, self-efficacy, psychological state, and excessive use of technology, as well as gender, age, personality, motivation, and socioeconomic status [6]. Situational factors include provocation, parental involvement, school atmosphere, and the surrounding environment [7]. These factors influence an individual’s internal state, leading to negative thoughts and actions that result in cyberbullying behavior. The current phenomenon shows that many teenagers experience depression, anxiety, discomfort, decreased school performance, social withdrawal, and even suicidal attempts due to cyberbullying [8].

In Indonesia, the number of teenagers who are victims of cyberbullying is reported to be 80%, with daily occurrences [9]. According to the United Nations Children’s Fund (UNICEF) report in 2016, cyberbullying victims in Indonesia reached 41–50% [10]. Forms of cyberbullying perpetrated include outing (spreading others’ secrets) at 36.25%, flaming (harsh messages) at 26.42%, and harassment (messages causing disturbances) [11]. Victims experience flaming (58.86%), harassment (45.72%), and cyberstalking (36.68%) [12]. The prevalence and impact of cyberbullying are increasingly concerning, particularly the emotional and psychological effects on adolescents.

Cyberbullying behavior significantly impacts adolescents’ emotional and psychological conditions, leading to negative emotional states such as sadness, anger, frustration, shame, and fear [13]. These unstable emotional and psychological conditions can contribute to mental health disorders. Adolescents’ physical, psychological, social, and academic well-being are affected, including physical complaints (e.g., headaches, sleep disturbances), psychological symptoms (e.g., depression, anxiety), and social withdrawal [14]. Such negative impacts require immediate attention, particularly from nurses, who play a pivotal role in addressing these issues. Nurses are uniquely positioned to prevent and manage bullying behaviors through primary healthcare efforts focused on promotive and preventive strategies [15].

**Fig. 1:**
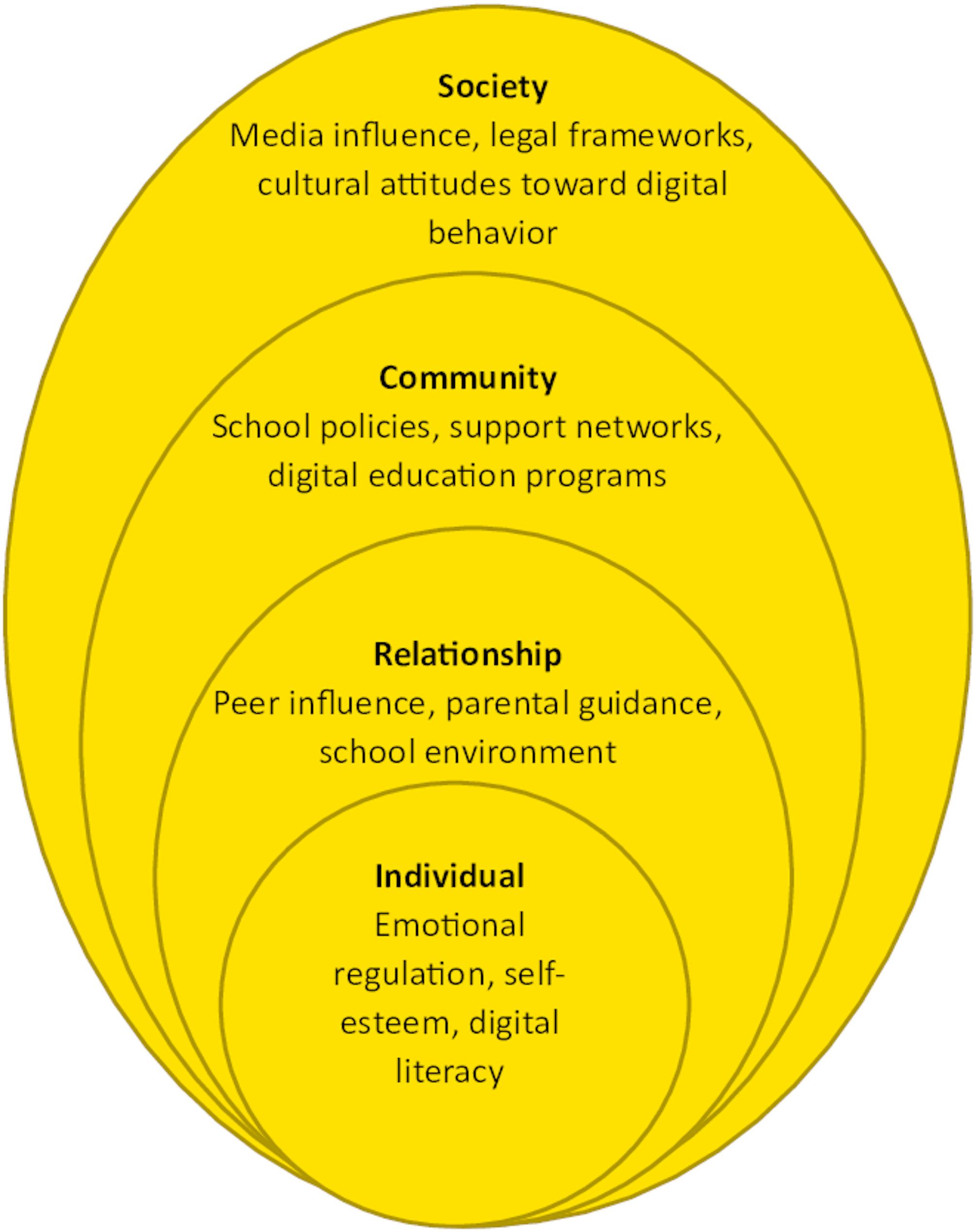
Social-ecological model

The social-ecological model of cyberbullying highlights the interplay of individual, relationship, community, and societal factors in the emergence of cyberbullying (Fig. 1) [16]. At the individual level, factors such as emotional regulation, self-esteem, and digital literacy influence adolescents’ susceptibility to cyberbullying. The relationship level includes peer influence, parental guidance, and school environment, which shape adolescents’ online interactions and responses to cyberbullying. The community level encompasses school policies, support networks, and digital education programs that can mitigate the risks and impacts of cyberbullying. At the societal level, broader factors such as media influence, legal frameworks, and cultural attitudes toward online behavior and digital ethics shape norms and responses to cyberbullying. Addressing these interconnected layers is essential for developing effective interventions [17, 18]. Furthermore, applying a holistic care model enables nurses to approach victims’ needs comprehensively, focusing on physical, psychological, social, cultural, and spiritual aspects. Utilizing a stress and coping framework provides insight into how nurses can support adolescents in developing adaptive coping mechanisms, reducing the impact of cyberbullying [19]. The behavioral change model further supports interventions by promoting positive behavior modifications among adolescents [20]. Additionally, understanding adolescent development perspectives is crucial in addressing the unique vulnerabilities of this age group.

Nursing interventions are crucial in mitigating the impacts of cyberbullying and improving adolescents’ well-being. Nurses can provide targeted health education to raise awareness about cyberbullying, its risks, and its consequences [21, 22]. They can also deliver specific interventions such as counseling programs, anger management sessions, and coping skills training, which directly address the psychological and emotional impacts of cyberbullying. Through their holistic, patient-centered approach, nurses assess not only the physical but also the psychological, social, and environmental aspects of adolescents affected by cyberbullying [23]. For instance, by involving parents and schools, nurses can foster a supportive network that helps adolescents build resilience and reduce vulnerability to cyberbullying.

Nurses are uniquely positioned to provide health education, such as counseling on anger management, promoting positive coping mechanisms, and educating adolescents and parents about cyberbullying prevention [24]. Effective nursing interventions not only address the immediate effects of cyberbullying but also contribute to long-term mental health resilience in adolescents [7, 25]. Understanding nurses’ perspectives and experiences in dealing with cyberbullying is essential to developing tailored interventions that address this pressing issue. While much research has focused on the prevalence and effects of cyberbullying, fewer studies have examined how mental health nurses perceive and respond to these challenges.

Despite the importance of the nursing role, little is known about how nurses perceive and manage cyberbullying cases. This research gap emphasizes the need to explore nurses’ first-hand insights, which could yield valuable information to improve nursing practice and patient outcomes. While existing studies focus on the effects of cyberbullying, few address the perspectives and experiences of mental health nurses. This study aims to fill this gap by investigating nurses’ perceptions and experiences in managing adolescent victims of cyberbullying, particularly in a referral hospital setting in West Java, Indonesia. By addressing this gap, the study contributes to the development of evidence-based interventions and policies to better support adolescents experiencing cyberbullying.

## Materials and methods

### Study design

This research was conducted using a qualitative approach with semi-structured interviews with mental health nurses who treat children and adolescents experiencing cyberbullying and work in a referral psychiatric hospital in West Java, Indonesia. The semi-structured interview guide was informed by a review of relevant literature and expert consultations, ensuring the questions aligned with the study objectives. Sample questions included topics such as the challenges nurses face when providing care, strategies they employ, and their perceptions of the impact of cyberbullying on adolescents.

The authors have a professional background in psychiatric nursing or a Ph.D. In mental health nursing. Our research team is made up of men and women, all of whom have research and publishing experience and are trained at an international level.

### Participant and setting

Participants in this study were mental health nurses caring for children and adolescents experiencing bullying or cyberbullying who were admitted to child and adolescent psychiatric wards. We used purposive sampling, which is recommended in qualitative research for selecting participants with familiarity and expertise in the topic area [26]. The inclusion criteria for this study required participants to be nurses working in inpatient psychiatric settings, holding at least a nursing degree, having a minimum of two years of experience in child and adolescent psychiatric care, and possessing experience in handling cases of cyberbullying. Nurses who were unavailable for interviews or unwilling to participate were excluded. Initially, 15 participants were recruited, all of whom were employed at a tertiary psychiatric hospital specializing in mental health rehabilitation programs for children and adolescents. However, three participants withdrew from the study, resulting in a final sample of 12 participants. Participants’ demographic information included years of experience (ranging from 2 to 15 years), educational backgrounds, and specialty areas within psychiatric nursing.

### Ethical considerations

Here is the revised version of your paragraph to reduce similarity while maintaining clarity and accuracy:

This study adhered to the ethical guidelines set by the Health Research Ethics Committee of the School of Nursing, STIKEP PPNI Jawa Barat, West Java (No. III/020KEPKSLE/STIKEP/PPNI/JABAR/VI/2022). Prior to participation, all participants received detailed information about the study’s objectives and were assured that they could withdraw at any stage without consequences. Written informed consent was obtained from each participant. To ensure confidentiality, interviews were conducted with respect for privacy, and all recorded data were anonymized using encrypted identifiers (P1–P12). Participation was entirely voluntary, and no physical or psychological risks were associated with the study.

### Data collection

The authors used semi-structured, in-depth interviews to examine the experiences of mental health nurses caring for children and adolescents who had experienced cyberbullying and were admitted to psychiatric wards in Indonesia. The interview guide was developed based on a literature review and validated by two experts in psychiatric nursing. Sample questions included: “What challenges do you encounter when caring for adolescents experiencing cyberbullying?” and “What strategies have you found effective in providing support to these adolescents?” Interview sessions commenced immediately after obtaining informed consent and securing permission to record participants’ voices. Data collection continued until saturation was reached, meaning no new themes emerged from further interviews. Each interview lasted approximately 45–60 min and was conducted in person. The study utilized various tools, including tape recorders, semi-structured interview guides, and field notes, while the researchers themselves played a central role in data collection. The interviews began with efforts to establish trust and rapport between the interviewer and the participant.

### Data analysis

This study utilized NVIVO 12 software (QSR International) to systematically classify and organize data obtained from participant citations and observations [27]. The analysis of interview data followed a six-step content analysis process:

1. Becoming familiar with the data by thoroughly reading and re-reading transcripts.
2. Systematically identifying and assigning initial codes to meaningful data segments.
3. Grouping related codes to identify potential themes.
4. Reviewing and refining themes to ensure consistency with the dataset.
5. Defining and labeling themes to establish their scope and meaning.
6. Compiling the final report, incorporating findings supported by relevant data [21].

For instance, codes such as “emotional support” and “collaborative care” were categorized under the broader theme of “holistic nursing interventions.”

### Triangulation and trustworthiness

The authors conducted validation tests on participants’ interview responses to ensure data accuracy. Data collection ceased once saturation was reached, indicating no emergence of new insights. This study employed triangulation across sources, techniques, and study duration [28]. To enhance data reliability, member checking and self-reflection were performed, while transferability was ensured by aligning the findings with the study’s objectives. Peer review, involvement of the thesis advisor as a reviewer, and adherence to research documentation, including the research report and manuscript—further reinforced the logical coherence and credibility of the study [29].

## Results

Characteristics of Participants Respondents in this qualitative study were 12 mental health nurses at a psychiatric hospital in West Java province, Indonesia. Initially, 15 participants were recruited; however, three participants withdrew, leaving 12 final participants. The age range of the respondents was 36–46 years. All respondents identified as Muslim. The gender distribution included 2 males (16.67%) and 10 females (83.33%). Educational qualifications included 2 respondents with postgraduate degrees in mental health nursing (16.67%) and 10 with undergraduate degrees in nursing (83.33%). Work experience ranged from 9 to 18 years (Table 1). Participant confidentiality was maintained by anonymizing data and assigning participant codes (P1-P12). Written informed consent was obtained prior to participation.

**Table 1:** Characteristics of participant

Number	Biological Gender	Religion	Education	Work experiences
P1	Female	Islam	Post-graduate	10 years
P2	Female	Islam	RN program	15 years
P3	Female	Islam	RN program	12 years
P4	Female	Islam	RN program	15 years
P5	Female	Islam	RN program	12 years
P6	Female	Islam	Post-graduate	9 years
P7	Male	Islam	RN program	16 years
P8	Female	Islam	RN Program	17 years
P9	Female	Islam	RN program	18 years
P10	Female	Islam	RN program	10 years
P11	Male	Islam	RN program	12 years
P12	Female	Islam	RN program	18 years

Themes This study identified five themes that align with the experiences of mental health nurses in managing cyberbullying cases among adolescents. Each theme is organized with subthemes and supported by quotes referenced in the text (e.g., see Quote 1) and summarized in Table 2 for further reference.

**Table 2:** Superordinate, subordinate themes, and participants’ quotations

Superordinate themes	Subordinate themes	Quotations
Types of Bullying	Verbal bullying	Q1: " My patient has experienced bullying, namely being looked down on by the parent’s job, usually the name of the parent, yes, that is based on the experience of my patient and my child” (P2)
		Q2: " My patient once told me something like this “don’t approach her because her economic status is not high enough, don’t hang out with her, her parents are poor. The patient also said that there were many hate comments on social media that insulted physically " (P10)
	Physical bullying	Q3: " Um, in my experience treating patients physically, the child moved schools 3 times, because he was uncomfortable due to being beaten by other teenagers " (P5)
		Q4: " my patient has experienced physical bullying, namely my ears were tweaked, then my buttocks were spanked by my friends” (P10)
Influencing Factors There is an increase in the skills and knowledge of nurses	Social Media As A Trigger	Q5: " The patient and my child told me that social media causes other youth to be free to bully through hate comments and teasing each other on social media " (P12)
	Unfriendly Parents	Q6: " Teenagers complain that their mothers don’t want to listen to their complaints. So when he was in 6th grade he was taken to the hospital, but his mother didn’t listen to his complaints.” (P9)
	Pattern Of Parenting	Q7: “Bully patients, on average, experience insecurity, those advantages, we train them so that they will feel happy too. But his parents also did not give comfort to their children” (P8)
		Q8: " Parents also lack attention, do not provide support to their children. As a nurse, I am confused about how to educate his family so that his family can accept him and the knowledge of bullying” (P7)
Obstacle of Cyberbullying Continuing Care	Lack of Communication	Q9: “So it’s difficult for me to talk to teenagers, because sometimes teenagers don’t open up during assessments. My child is also at home, so he doesn’t communicate with me” (P7)
		Q10: “I have also experienced something similar, namely handling bullying victims but the patient doesn’t want to talk” (P10)
	Difficult For the Perpetrator to Intervene	Q11: “Most of these patients are victims of cyberbullying, so actually to prevent bullying from occurring, we also need to intervene with the perpetrators. It’s just that we rarely find bullies who are brought in for therapy“(P5)
	Mood Of Nurses	Q12: “Victims of bullying need more attention, right? Meanwhile, my mood as a nurse is not good, so I have to manage my mood so that I don’t transfer negative feelings to patients. This also makes it difficult for me to deal with patients who are victims of bullying” (P4)
	Need Specific Of Competences	Q13: “Teenagers need supervision so that their adolescent development stages can be fulfilled. So that teenagers who are victims of bullying certainly need special interventions that focus on bullying problems and also the stages of adolescent development “(P5)
	Special Approach For Children And Adolescents	Q14: “Yes, I agree, that teenage patients need a special approach to meet their developmental stages. Apart from that, we also need the latest communication and information skills to adapt to their generation” (P6)
	Tools For Assessment	Q15: “Sometimes I find it difficult to conduct an assessment on victims of bullying, because there are so many impacts that there must be short assessment tools to find out the impact of bullying” (P1)
	Managerial Aspect For Victims Of Cyberbullying	Q16: “Besides that, I think a special flow is needed to handle cyberbullying victims. The problem is I’m also confused about what the flow is like, if there is already a flow of handling it seems like it will make it easier for us and also victims of bullying” (P12)
Nursing Intervention	Give a Special Coping	Q17: “Adolescents who are victims of bullying need intervention to improve their coping with bullying. So these teenagers can be resilient to the effects of bullying and get out of their problems.” (P10)
	Need to Increase Self-Esteem	Q18: “Many victims of bullying also experience insecurity, are embarrassed to speak and don’t want to look at me when they are chatting, so I always try to give positive affirmations to increase the victim’s self-confidence” (P2)
	Attention for Victims and Perpetrator	Q19: “Because there are victims of bullying who don’t get attention from their parents, they usually need more attention from nurses, so they feel they are not alone in facing their problems” (P3)
Specific Experiment	Arises from The Mother’s Instinct	Q20: “I have to improve my mother’s instinct as a nurse and parent in paying attention to my child, is he being bullied or not?” (P11)

## Theme 1: Types of bullying

The nurses identified two primary types of bullying: verbal bullying (e.g., derogatory comments on social media) and physical bullying (e.g., physical altercations such as hitting). Verbal bullying was the most frequently encountered form, with significant emotional impacts on adolescents (see Quote 1 and Quote 2). Physical bullying, though less common, often led to visible injuries and required immediate intervention (see Quote 3 and Quote 4).

### Theme 2: Influencing factors three key factors exacerbating cyberbullying were identified

- Social Media as a Trigger: Platforms were noted as environments where bullying behaviors, such as negative commenting and teasing, escalate (see Quote 5).
- Unfriendly Family Dynamics: Adolescents who lacked family support were less likely to disclose their bullying experiences (see Quote 6).
- Parenting Patterns: Ineffective supervision of adolescents’ social media use and lack of awareness of emotional needs contributed to unresolved bullying (see Quotes 7–8). These factors influenced nurses’ strategies to involve families in the intervention process.

**Theme 3: Obstacles in Managing Cyberbullying This theme focuses on challenges faced by nurses**,** categorized into the following subthemes**:

- Communication Barriers: Adolescents often struggle to articulate their experiences, requiring nurses to build trust and rapport (see Quotes 9–10).
- Intervening with Perpetrators: Nurses expressed difficulty in addressing bullies due to the absence of clear protocols or guidelines (see Quote 11).
- Need for Specialized Competencies: Participants highlighted a need for training in age-specific communication, internet trends, and understanding youth culture (see Quotes 13–14).
- Assessment Tools: The lack of standardized tools for evaluating bullying impacts hindered the development of effective care plans (see Quotes 15–16). The statement “we have to adjust to the psychological condition of the victims” has been clarified to reflect nurses’ experiences in tailoring care to adolescents’ emotional states.

### Theme 4: Nursing interventions key nursing interventions included

- Building Coping Mechanisms: Nurses tailored coping strategies to the developmental stage and specific needs of the victims (see Quote 17).
- Enhancing Self-Esteem: Structured interventions, such as confidence-building activities, helped adolescents regain self-assurance (see Quote 18).
- Providing Holistic Attention: Ensuring victims felt supported and included was a core priority. Nurses also extended attention to perpetrators to prevent repeat incidents (see Quote 19).

### Theme 5: Holistic approaches through “Mother’s instinct”

Nurses described their empathetic and intuitive approach as a “mother’s instinct,” enabling them to address adolescents’ needs holistically. This included attention to physical, psychological, social, spiritual, and cultural dimensions. Collaboration with families and schools was emphasized to create a comprehensive support system (see Quote 20).

## Discussion

This study explored the experiences of mental health nurses in caring for children and adolescents who experienced bullying or cyberbullying and were hospitalized in psychiatric wards. The findings are discussed in relation to key themes, relevant literature, and implications for nursing practice and policy.

### Types of bullying

This study found that nurses identified two main types of bullying: verbal and physical bullying. Verbal bullying, including name-calling, teasing, and threatening, was the most common form encountered. This aligns with previous research that reported verbal bullying as the dominant type of bullying (66.5%) compared to physical bullying (32.0%) and social bullying (30.6%) [30]. This study extends prior findings by showing that nurses often observed a transition from physical to cyberbullying among adolescents, consistent with reports that physical bullying has decreased while cyberbullying has become more prevalent, particularly among younger adolescents [31]. Interestingly, verbal bullying trajectories are associated with moral disengagement and gender differences, with face-to-face bullying being more common among girls [32]. This study highlights the importance of addressing these nuances, as they inform tailored nursing interventions that account for gender and developmental differences [33].

### Influencing factors

The findings identified social media, unfriendly families, and parenting styles as key factors influencing bullying experiences. Social media use was linked to cyberbullying, as prolonged exposure increased opportunities for online harassment [34]. This aligns with prior studies demonstrating that social media amplifies victimization and distress among adolescents [35]. Social media can mediate the relationship between adolescents’ perceptions of bullying at school and their feelings of distress. However, this mediating effect is not observed when considering students’ perceptions of the frequency of cyberbullying and adolescent distress. Bullying cases can interfere with coping which can lead to positive cognitive impairment that will affect the healing process from symptoms of stress due to bullying. Next, we present evidence that social media rumination is experienced by adolescents, and has a distinct influence on adolescents [35]. However, this study provides new insights by showing that nurses perceive social media as a medium that mediates bullying-related distress and complicates adolescents’ coping mechanisms.

Unfriendly family dynamics were found to exacerbate bullying outcomes, consistent with studies emphasizing the role of ineffective family communication and lack of parental support [36]. While parental support may not always buffer the effects of peer victimization, it contributes to adolescents’ overall well-being by reducing depressive symptoms [37]. Nurses in this study emphasized the need to involve families in interventions, particularly those with democratic parenting styles, as these are associated with better outcomes [38].

Parenting styles that are excessively rigid or overly permissive carry a high risk of contributing to bullying behavior in adolescents. To prevent this risk, parents are encouraged to adopt an authoritative parenting style [39]. Based on studies presented in seminars on cyberbullying and the Triple P e-Parenting program, this approach has been proven effective in increasing nurses’ awareness, as parents, of the occurrence of cyberbullying among adolescents. By considering the experiences of parents with children affected by cyberbullying, nurses can better understand parental attitudes and effective parenting strategies for its prevention. One such approach is the Positive Parenting Program (Triple P), which plays a significant role in enhancing parenting practices, addressing children’s behavioral problems, and improving overall family well-being [24].

### Obstacles and difficulties

Nurses reported internal and external challenges in managing bullying cases. Internal barriers included communication difficulties, the need for specialized competencies, and the psychological impact on nurses, which aligns with research highlighting workplace stress and the emotional toll of psychiatric nursing [36]. External barriers, such as the lack of assessment tools and organizational support, are consistent with findings that structural inadequacies hinder nurses’ ability to provide effective care [38].

The absence of clear guidelines for addressing bullying perpetrators was a significant challenge. Previous studies have called for frameworks to guide interventions, particularly those addressing the emotional states and distorted thinking patterns of bullies [35]. This study underscores the need for comprehensive training and resources to equip nurses with the skills and tools required to address these issues effectively.

### Nursing interventions

Nursing interventions identified in this study focused on building coping mechanisms, enhancing self-esteem, and providing holistic attention to both victims and perpetrators. These interventions align with evidence-based approaches, including prevention programs, peer group activities, and resilience-building initiatives [40]. Nurses emphasized the importance of tailoring interventions to adolescents’ developmental stages and emotional needs, reflecting findings that effective coping strategies are influenced by perceptions of problem-solving abilities and emotional control [41, 42]. This study highlights the critical role of emotional-focused and problem-focused coping strategies in addressing cyberbullying impacts. It also emphasizes the importance of addressing the emotional drivers of bullying behavior, such as anger and cognitive distortions, to prevent recurrence [43, 44].

The socio-ecological model of bullying takes a holistic view of the influence of the environment on individual development, considering factors that interact at multiple levels, from the micro (individual), meso (interpersonal relationships), to macro (social structure and policy) [17]. The application of this model allows nurses to identify the various factors that play a role in adolescent bullying experiences, both from the individual side (e.g., level of psychological vulnerability), social interactions (such as peer and family dynamics), and the broader environment (including school policies and social norms) [19]. The experience of bullying is the result of a complex interaction between these factors, nurses can design interventions that not only focus on healing the victimized individual, but also improve or change the social environment that can facilitate or prevent bullying. Nurses can use this model to design interventions that involve increasing social awareness in schools, strengthening positive communication patterns within families, and advocating for policies that support bullying prevention [45].

In nursing practice, the social-ecological model serves as a guide to create more comprehensive and sustainable interventions in dealing with bullying cases. This approach allows nurses to identify the various layers that contribute to the problem of bullying, considering not only the experiences of victims, but also the role of bullies, as well as the environment that shapes the interaction [17]. Nurses can design training programs for parents with the aim of introducing supportive parenting, as reflected in the Triple-P (Positive Parenting Program), to increase parents’ awareness of the influence of their behavior on the likelihood of bullying [24]. On the other hand, nurses can also collaborate with schools to create a more inclusive environment, by facilitating social education programs that teach students interpersonal skills and empathy. Nurses can engage in policy advocacy to promote stricter regulation of the use of social media that can exacerbate online bullying [46].

Nurses worldwide report that bullying, both physical and verbal, remains a major problem faced by adolescents, with significant psychological impacts. There is a greater emphasis on school-based anti-bullying programs and interventions by mental health professionals in educational settings [42]. While there is a similar focus on the need for social support-based interventions, this study suggests that in Indonesia, families play a more significant role in bullying interventions, particularly with a more authoritative parenting approach. In this regard, family involvement plays a more important role in addressing the impacts of bullying compared to Western countries where peer support and institutional policies are more heavily relied upon [47].

Another uniqueness of Indonesian mental health nursing is seen in the way nurses adapt their interventions to local cultural values. In Indonesia, a strong collectivist culture and the dominant role of the family in adolescents’ lives significantly influence the approach to mental health care. Nurses in Indonesia often face challenges in integrating family-based interventions with social norms that emphasize harmony and respect for family authority [23]. Individual independence, reflected in self-based interventions and more structured psychotherapeutic approaches [48]. The findings of this study identified that authoritative parenting patterns demonstrated by parents were positively associated with reduced bullying impact, reflecting the importance of effective communication and emotional attention in the Indonesian family context.

In addition, these findings also highlight the differences in bullying management between developed countries and Indonesia in terms of limited resources and infrastructure to support bullying prevention programs. Nurses have better access to specialized training, standardized assessment tools, and interprofessional collaboration in handling bullying cases. In contrast, in Indonesia, nurses often face obstacles related to the lack of appropriate assessment tools, limited training, and adequate organizational support. Therefore, although the basic principles of mental health nursing to address bullying may be similar, the different structural challenges and resources between Indonesia and Western countries require adjustments in the development of policies and intervention programs that are more appropriate to the local context.

### Holistic approaches through “Mother’s instinct”

Nurses described using “mother’s instinct” as a natural coping mechanism, demonstrating empathy and emotional recognition when caring for adolescents who experienced bullying. This finding is consistent with research indicating that empathy enhances ethical and compassionate care. Similarly, a review study concluded that nurses base their interventions on empathy and emotional recognition, which involve providing companionship to clients, engaging in conversations with adults, and redirecting attention to promote well-being [49]. Empathy helps nurses become more sensitive and act ethically in dealing with child clients, it helps nurses in providing soothing care [50]. But this needs to be considered because nurses also need to consider their professional boundaries, even though they take on empathetic and nurturing roles like parents, but nurses need to provide clear boundaries between professional and personal relationships. However, nurses must balance their nurturing roles with professional boundaries to maintain effective and ethical care. This study contributes to the literature by highlighting the importance of emotional intelligence and empathy in psychiatric nursing practice.

### Study limitations

This study had several limitations. First, the small sample size and single-site setting may restrict the generalizability of the findings. Conducting sampling across multiple regions could offer a more comprehensive understanding of nurses’ experiences. Second, the study focused exclusively on nurses’ perspectives, excluding insights from patients and other healthcare professionals, which may have enriched the findings. Future research should address these limitations by including diverse perspectives and larger, more varied samples. Additionally, future studies could explore adolescent perspectives on nurses’ interventions to better understand their effectiveness and acceptability. Further research could also evaluate the long-term impact of these interventions, develop digital-based nursing interventions, and examine the influence of cultural and social factors on intervention outcomes.

### Implications for nursing practice

The findings of this study have important implications for nursing practice and support services aimed at adolescents affected by bullying and cyberbullying:

1. **Training and Resources**: Comprehensive training programs should be developed to equip nurses with the skills and knowledge needed to address bullying effectively. These should include frameworks for assessing and intervening with both victims and perpetrators, as well as strategies for understanding the role of social media in mediating distress and coping mechanisms among adolescents.
2. **Family Engagement**: Nurses play a crucial role in engaging families, particularly those with democratic parenting styles, to enhance support for adolescents. The study highlighted that unfriendly family dynamics and ineffective communication can exacerbate bullying outcomes, while supportive, authoritative parenting can help mitigate these effects. Nurses should identify specific parenting styles, guide parents on how to provide positive support, and assess the effectiveness of family involvement in the intervention process. Integrating programs such as the Triple-P (Positive Parenting Program) can help parents address problematic behaviors and improve their children’s well-being. Nurses should actively engage with families, providing them with strategies to strengthen family support systems for better outcomes in adolescents.
3. **Organizational Support**: Healthcare organizations should provide clear guidelines, assessment tools, and adequate resources to support nurses in managing bullying cases effectively, particularly with regard to the role of social media and family dynamics.
4. **Interdisciplinary Collaboration**: Nurses should collaborate with schools, families, and mental health professionals to create a holistic support system for adolescents, ensuring that all aspects of the adolescent’s well-being are addressed in a coordinated and comprehensive manner.

## Conclusion

This study explored the experiences of mental health nurses caring for children and adolescents hospitalized due to bullying or cyberbullying. The findings revealed the predominant role of verbal and physical bullying, with social media use, family dynamics, and ineffective parenting exacerbating the issue. Despite challenges such as communication barriers and the lack of assessment tools, nurses demonstrated adaptive strategies, including enhancing coping mechanisms and providing holistic care. The concept of “mother’s instinct” emerged as a key coping mechanism for nurses. This study emphasizes the importance of empathy, emotional intelligence, and comprehensive intervention strategies in nursing practice. The findings highlight the need for structured training, family engagement, and organizational support. Future research should focus on developing interventions to enhance nurses’ competencies in managing bullying cases across diverse settings.

This study explored the experiences of mental health nurses in caring for children and adolescents who experienced bullying or cyberbullying and were hospitalized in psychiatric wards. The findings revealed several key themes that highlight the critical role of nurses in addressing the multifaceted challenges posed by bullying. Nurses identified verbal and physical bullying as the predominant forms, with verbal bullying being more frequent and transitioning into cyberbullying among adolescents. Influencing factors such as social media use, unfriendly family dynamics, and ineffective parenting were found to exacerbate bullying experiences. Nurses also faced significant internal and external obstacles, including communication barriers, the absence of assessment tools, and organizational limitations, which impacted their ability to provide effective care.

Despite these challenges, nurses demonstrated adaptive strategies such as enhancing adolescents’ coping mechanisms, improving self-esteem, and providing holistic care. The unique concept of “mother’s instinct” emerged as a natural coping mechanism for nurses, allowing them to empathize deeply with their clients while maintaining professional boundaries.

This study highlights the critical role of nurses in addressing adolescent bullying and cyberbullying, emphasizing the need for structured training in empathy and emotional intelligence within nursing education. The findings also underscore the importance of healthcare policies that support interdisciplinary collaboration and institutional frameworks to equip nurses with effective intervention strategies. Strengthening these aspects in nursing curricula and policy development will enhance nurses’ competencies and contribute to a more comprehensive approach to adolescent mental health.

## Electronic supplementary material

Below is the link to the electronic supplementary material.

Supplementary Material 1

## Data Availability

The data that support the findings of this study are available from the corresponding author, [IY], upon reasonable request.
