# Supplementary material for: Experiences of mental health nurses who give nursing intervention among child and adolescent with cyberbullying: a qualitative study
Source: BMC Nurs. 2025 May 14;24:527. doi: 10.1186/s12912-025-03182-x (PMC12080159; doi:10.1186/s12912-025-03182-x)
Supplement: Supplementary file 1 — Supplementary Material 1 [file 12912_2025_3182_MOESM1_ESM.docx]

**Revised Guidelines for Semi-Structured Interview Questions**

1. Based on your experience, what type of bullying is most commonly experienced by patients or the families of your patients?
2. Have you ever witnessed or intervened in cases of verbal bullying, either online or in person? If so, how did you address it in your practice?
3. Have there been cases of physical bullying involving children or teenagers around you? If so, how do you typically intervene in such situations?
4. How do you perceive the impact of social media on bullying cases, and how do you address this in your interventions with patients?
5. How do parental attitudes influence the emotional condition of teenagers who are victims of bullying, and how do you involve parents in your interventions?
6. What specific communication strategies do you use when engaging with victims of bullying, particularly in cases of cyberbullying?
7. How do you approach cases where the bullying perpetrator is difficult to engage in therapy or intervention?
8. Does your emotional state or mood affect your interactions with bullying victims? If so, how do you manage this in a professional setting?
9. In your view, what specific competencies should a nurse have to effectively address bullying issues in children and teenagers?
10. How do you tailor your approach to address the psychological needs of children and teenagers who have experienced bullying?
11. Can you describe any specific interventions or approaches that have been effective in supporting bullying victims during therapy or counseling?
12. How do you help bullying victims rebuild their self-esteem and confidence? Can you provide an example of a successful intervention?
13. How do you provide extra attention to bullying victims to help them feel supported? Can you describe a situation where this approach was particularly successful?
14. How do you ensure that your approach to treating bullying victims is holistic, including physical, psychological, social, spiritual, and cultural aspects?
